# Tailored elastic surface to body wave Umklapp conversion

**DOI:** 10.1038/s41467-020-17021-x

**Published:** 2020-06-29

**Authors:** Gregory J. Chaplain, Jacopo M. De Ponti, Andrea Colombi, Rafael Fuentes-Dominguez, Paul Dryburg, Don Pieris, Richard J. Smith, Adam Clare, Matt Clark, Richard V. Craster

**Affiliations:** 1 Department of Mathematics, Imperial College London, London, SW7 2AZ UK; 2 Department of Civil and Environmental Engineering, Politecnico di Milano, Piazza Leonardo da Vinci, 32, 20133 Milano, Italy; 3 Department of Mechanical Engineering, Politecnico di Milano, Via Giuseppe La Masa, 1, 20156 Milano, Italy; 4 Department of Civil, Environmental and Geomatic Engineering, ETH, Stefano-Franscini-Platz 5, 8093 Zürich, Switzerland; 5 Optics and Photonics, Faculty of Engineering, University of Nottingham, Nottingham, NG7 2RD UK; 6 Advanced Component Engineering Laboratory (ACEL), Faculty of Engineering, University of Nottingham, NG7 2RD Nottingham, UK; 7 Department of Mechanical Engineering, Imperial College London, London, SW7 2AZ UK; 8 UMI 2004 Abraham de Moivre-CNRS, Imperial College London, London, SW7 2AZ UK

**Keywords:** Engineering, Physics

## Abstract

Elastic waves guided along surfaces dominate applications in geophysics, ultrasonic inspection, mechanical vibration, and surface acoustic wave devices; precise manipulation of surface Rayleigh waves and their coupling with polarised body waves presents a challenge that offers to unlock the flexibility in wave transport required for efficient energy harvesting and vibration mitigation devices. We design elastic metasurfaces, consisting of a graded array of rod resonators attached to an elastic substrate that, together with critical insight from Umklapp scattering in phonon-electron systems, allow us to leverage the transfer of crystal momentum; we mode-convert Rayleigh surface waves into bulk waves that form tunable beams. Experiments, theory and simulation verify that these tailored Umklapp mechanisms play a key role in coupling surface Rayleigh waves to reversed bulk shear and compressional waves independently, thereby creating passive self-phased arrays allowing for tunable redirection and wave focusing within the bulk medium.

## Abstract (web summary)

Umklapp scattering, an effect that has been conventionally studied in phonon systems in quantum transport, is studied here in an elastic system. The authors demonstrate mode conversion from surface Rayleigh waves into bulk waves that have uniquely tunable properties.

## Introduction

The Umklapp, or flip-over process first hypothesised by Peierls^1^ is conventionally concerned with describing phonon–phonon scattering to explain thermal conductivity at high temperatures, and has a rich history in the quantum theory of thermal transport^2,3^. Concepts based around the Umklapp process are not traditionally incorporated in areas of wave physics concerning designs of elastic metasurfaces; deep elastic substrates support surface Rayleigh waves that propagate along the surface, often over large distances, which are an essential component of, for instance, surface acoustic wave microfluidic devices^4^, acoustic microscopy^5^, at small-scales and of seismic wave and groundborne vibration propagation at the geophysical scale^6^. An isotropic, and homogeneous, elastic medium supports two types of bulk waves: compressional, P, and shear, SV and SH, waves polarised vertically and horizontally that propagate with different wavespeeds *c*_p_ and *c*_s_ with *c*_p_ > *c*_s_^7^ with the Rayleigh wavespeed *c*_r_ slower than both.

Recently emerging ideas in graded metamaterial arrays and so-called rainbow-trapping devices have presented novel ways to manipulate wave propagation. Taking advantage of ideas that emerged in optics around slow-light devices and optical “rainbow” trapping^8^ have in turn motivated tailored designs of graded Helmholtz resonator arrays in acoustics^9^, and their analogues in water waves^10^, to slow the array-guided waves and trap the waves at different spatial positions, with application to broadband sound absorbers^11,12^. In almost all wave regimes where these graded systems are designed, Umklapp effects have been neglected.

Neither acoustic nor electromagnetic waves have the additional complications of elasticity, that is, having both shear and compressional wavespeeds, mode coupling at interfaces and Rayleigh surface waves; this presents an opportunity as the additional degree of freedom in elasticity can be exploited. Elastic graded resonator arrays use rods or beams, whose length determines the resonance frequency, and grading creates a metawedge^13^. Trapping and slow-wave phenomena occur but now with the additional physics of mode conversion from the Rayleigh wave into a forward-propagating shear wave in the bulk as confirmed experimentally^14^. The trapping phenomena has potential for energy harvesting^15^; the metawedge provides spatial segregation by frequency thereby amplifying elastic energy at specific resonators that can be coupled to piezoelectric patches^16,17^.

The physics of a graded structured array can also be interpreted in terms of phase changes, induced by changes in the array elements, as the wave transits the array. In this vision, a graded array acts as a self-phased array and one can induce backward-directed radiation from an array (Supplementary Note 5, Supplementary Fig. 4). In the context of a model for flexural waves on thin elastic plates, a simple scalar model, a graded line array has been shown^18^ to create focusing and flat-lensing effects, that emulate negative refraction on a line and Pendry–Veselago flat lenses. Although motivational, the thin plate model also lacks the multiple wavespeeds of the deep elastic substrate, and the array-guided wave only exists below the sound-line, i.e. in the first Brillouin zone.

**Fig. 1:**
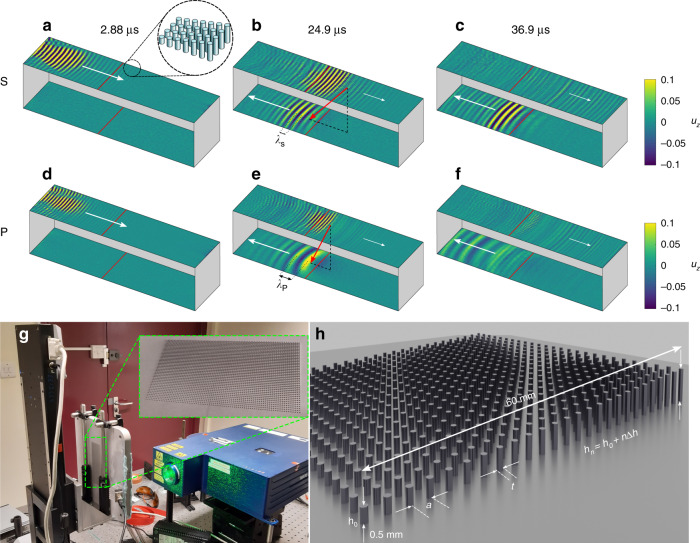
Experimental results. Snapshots in time of temporal–spatially filtered scans along top and bottom surfaces observing S conversion (**a**–**c**) and P conversion (**d**–**f**), filtered between 1.1–1.2 and 1.45–1.55 MHz, respectively, normalised to the maximum of displacement of the top surface (*u*_*z*_). Solid red lines show where graded array begins, with increasing rod height in the direction of wave propagation on the top surface. The reversed conversion is clear; the forward propagating surface wave on the top surface is reverse converted into the bulk and is seen to excite reversed propagating surface waves on the bottom surface. The measured angles of reversed conversion,  −131.8^∘^ and  −106.9^∘^ for S and P, respectively, match the predicted angles from Fig. 3, with the separate polarisations evident from the difference in excitation wavelength on the bottom surface, marked *λ*_S_, *λ*_P_. The experimental setup is shown **g**, as is a schematic of array geometry **h** detailing the rod diameter, *t*, periodicity *a* and grading through the changes in height of the *n*th rod, *h*_*n*_, as *h*_*n*_ = *h*_0_ + *n*Δ*h*. Fabrication details are given in the “Methods” section, with the array parameters given in Supplementary Table 1.

In this article, by taking an elastic half-space, patterned by a graded resonant array of rods (Fig. 1), we show that, one can obtain mode conversion from surface waves to compressional, P waves, and not just to shear, S waves in the bulk. Furthermore, this can be directed backward, and used to create focusing, and these features are due to Umklapp scattering from outside the first Brillouin zone. These striking results are confirmed experimentally from the predictions made by the theory and by simulation. The elastic wave system, having distinct wave types with different wavespeeds, is imbued with richer physics than acoustic/electromagnetic systems and this yields a greater degree of flexibility and the opportunity for novel effects; in elasticity we have two distinct sound-lines, also as the Rayleigh surface wave is, unlike spoof surface plasmons, not induced by periodic geometrical changes it is not confined to lie below the sound-line nor is it confined to be within the first Brillouin zone.

## Results

### Design paradigm

**Fig. 2:**
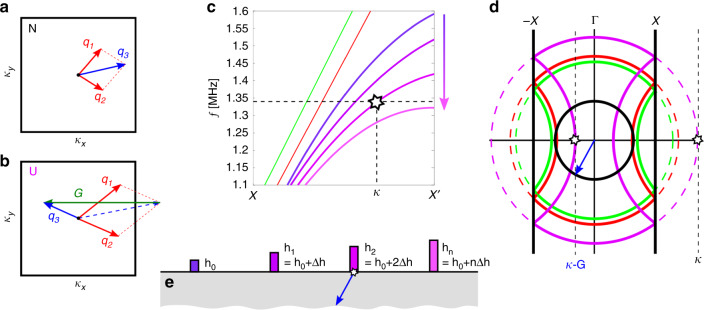
Theoretical prediction from local dispersion curves. **a** Wave vector scattering in the first Brillouin zone showing conventional definitions of an N-process and a U-process **b**; the ‘flip-over' description is only strictly true for collinear scattering^19^. **c** Longitudinal dispersion curves within second Brillouin zone; *X* marks the edge of the first Brillouin zone, whilst $X^{\prime}$ marks the edge of the second Brillouin zone. Each dispersion curve represents the second longitudinal mode for a perfectly periodic array of rods, with the fixed heights with R(S) sound lines shown in red(green). **d** Isofrequency contours of rod where last longitudinal mode supported, after which an effective bandgap is reached, when exciting at a frequency of 1.340 MHz. At this position U-processes are preferential to a change in rod motion and, resulting in an effective reversed wavenumber ***κ*** − **G**, capable of mode converting into a P wave (black isofrequency contour, shown in full dispersion curves in Supplementary Fig. 1). **e** Graded array schematic colours corresponding to the curves in **c**.

When operating outside the first Brillouin zone, concepts of crystal momentum become important^19^. The conventional definition of an Umklapp process, or U-process, is elucidated in Fig. 2a, b, whereby the resultant wavevector of a scattering process within a periodic crystal lies outside the first Brillouin zone; promotion of a backward-propagating phonon results through crystal momentum transfer, since the wave vector, **q** is defined modulo **G**, i.e. up to a reciprocal lattice vector. The textbook distinction between normal (N-processes) and U-processes is then given by

1 $${{\bf{q}}}_{1}+{{\bf{q}}}_{2}-{{\bf{q}}}_{3}=\left\{\begin{array}{ll}{\bf{0}}&{\rm{N}}{\hbox{-}}{\rm{process}},\\ {\bf{G}}&{\rm{U}}{\hbox{-}}{\rm{process}}.\end{array}\right.$$

where the **q**_*i*_ are the wave vectors in Fig. 2a, b. We stress that these mechanisms do not violate momentum conservation^1^; unlike in conventional graded structures we are not considering only the true momenta of interfering waves (phonons) within a crystal, but taking advantage of the momentum of the system as a whole. Nuances of this simplistic definition arise due to the apparent interchangeability of N-process and U-process through altering the primitive cell, and the associated (quasi)momentum conservation^19–21^. The distinction is achieved throughout by analysing the dispersion curves of the locally periodic elements, along with simplified isofrequency contours (Supplementary Figs. 1 and 2). Recently the importance of the Umklapp process has been solidified for electron–electron scattering through experimental verification, highlighting the fundamental role it plays in electrical resistance in pure metals^22^, along with the utility to probe electronic structures^23^. Further to this, new breeds of entirely flat lens devices have been theorised which incorporate Umklapp-scattering processes for surface waves on dielectric substrates^24^ by virtue of abrupt, not adiabatic, gradings.

**Fig. 3:**
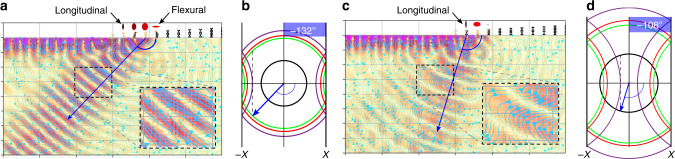
Simulations of reversed conversion by Umklapp scattering. **a** S conversion for excitation at *f* = 1.2 MHz, predicted using isofrequency contours shown in **b**. **c** P conversion for excitation at *f* = 1.45 MHz, predicted using isofrequency contours shown in **d**. In each case at the position of conversion is determined by the change from longitudinal to flexural motion of neighbouring rods. The ratio of the relative magnitude of longitudinal to flexural motion is shown by the ellipses above the rods. The angle of conversion, computed directly from the simulated field transformed in the wavenumber space, matches the predictions from the isofrequency curves of the last rods supporting the longitudinal mode, similarly to Fig. 2c. The difference between the converted wave types (S and P) can be seen clearly from the streamline analysis. Wavenumber analysis on the simulated data also confirmed the two different velocities ~3000 m s^−1^ for S and 6000 m s^−1^ for P. Array parameters are detailed in Supplementary Table 1.

Undeterred by its prevalence in quantum, discrete systems we demonstrate the efficacy of U-processes which can preside over purely passive, classical, continuous elastic devices. The extent of this mechanism is often not considered in the wide range of structures based on (locally) periodic material systems, and indeed neglected in elasticity theories^25^. Incorporating this effect is achieved by utilising the existence of surface waves outwith the periodic structure, and marrying the transition of such a wave to the excited wave within a graded structuring. By doing so, striking reversed conversion into S and P waves can be achieved, and controlled, as predicted in Fig. 3 and experimentally verified in Fig. 1. We present here the design methodology for structures capable of the conversion of Rayleigh waves directly into both S and P waves independently, by utilising the dispersion curves and isofrequency contours of perfectly periodic arrays of rods. Due to the adiabatic changes of the array parameters the global spatial properties of the array are determined by the locally periodic structures; the dispersive properties of the array at a given position are inferred from the periodic dispersion curves of the constituent element at that position^11,13^.

Shown in Fig. 2c, are the dispersion curves for the longitudinal motion of rods with fixed heights within the second Brillouin zone. These curves are obtained through an averaging process of the fully polarised dispersion curves (Supplementary Note 2, Supplementary Fig. 1), since incident R-waves excite a superposition of longitudinal and flexural rod motion^26^. Utilising modes in this region of reciprocal space, i.e. outwith the first Brillouin Zone, is somewhat counter-intuitive, in that the waves which appear above the light-line in an irreducible representation of the Brillouin Zone are traditionally ignored when considering spoof surface plasmons^27^, which are the closest electromagnetic analogue to the Rayleigh wave. The behaviour of the adiabatically graded array, as shown in Fig. 2e is predicted from these locally periodic curves. For a given frequency, longitudinal motion is preferentially supported by a number of rods; at some rod height however, there is a transition in preferred rod motion from longitudinal to flexural (or vice versa). This is seen as an effective band gap in Fig. 2c for the fourth rod, where U-processes dominate; the transfer of crystal momentum results in an effective reversed wavenumber, ***κ*** − **G**. Depending on the operational frequency, this can lie within the isocircle of the free S or P body waves; reversed conversion is achieved by conservation of the tangential component of the wavevector. Figure 2d shows the prediction of this angle by inspection of the last supported longitudinal mode (corresponding to the third resonator). The projected dispersion curves show the resultant wave vector inside the first Brillouin zone, as a result of transfer of crystal momentum, by the white star corresponding to that in Fig. 2c. The wave can hybridise with a reversed P wave by the phase matching with the isofrequency circle of the P wave, shown in black. A similar analysis can be carried out for conversion into S waves, by operating at a lower frequency. In this way, either longitudinal or flexural rod motion can excite either S or P reversed waves by the inspection of the transition from one dominant rod motion to another.

When operating at higher frequencies, corresponding to wavevectors outside the first Brillouin zone, U-processes occur regardless of any grading; energy is continually shed along the array (Supplementary Note 3, Supplementary Fig. 1c), resembling an elastic leaky wave antenna^28^. This can be manufactured to take place at a desired position by either adiabatic or abrupt gradings^24^. A similar effect is observed when exciting along the opposite array direction; the grading experienced is then from high to low rods. In this case, since the effective bandgap is not a true bandgap, propagation occurs through the array with Umklapp processes taking place all along the array (Supplementary Note 6, Supplementary Figs. 5 and 6).

### Simulation

Within this simple metawedge design paradigm lies many degrees of freedom: the position, angle and wave-type of reversed conversion, all allow a tailored conversion which simply relies upon a change in primary mode behaviour between neighbouring rods, within a higher Brillouin Zone. Demonstrated in Fig. 3, modelled using SPECFEM^29^, is the reversed conversion into S and P modes via the transition from longitudinal to flexural rod motion for an array with parameters detailed in Supplementary Table 1. The ellipses above the rods convey the ratio of the two dominant rod behaviours via their semi-minor and major axis. The angle of conversion comes from the conserving the tangential component of the wavevector, determined via the isofrequency contours of the rod prior to the change in rod motion (Supplementary Fig. 2), showing S conversion for 1.2 MHz and P conversion for 1.45 MHz.

### Experimental verification

Simulation results are confirmed experimentally in a 1.8 cm-thick slab of aluminium patterned using 3D printing, with a graded array of aluminium microrods on the surface (Fig. 1, Supplementary Fig. 6). A laser adaptive photorefractive interferometer scans the surface of the aluminium sample providing a reading of the displacement field in the out of plane direction *u*_*z*_. The block is attached to a moving platform. Pure Rayleigh waves are generated by an ultrasonic transducer attached at the surface of the aluminium sample (see “Methods” section for details on the experimental set-up). To enhance the visualisation of the conversion spatial and frequency filters have been applied. Time series have been bandpassed between 1.1–1.2  and 1.45–1.55 MHz for S and P conversion, respectively. The wavefield scans have been filtered selecting wavevectors pointing towards (away) from the resonators for the top (bottom) surface. This procedure mainly remove echoes from the boundaries as well as leakage from the transducer. The wavelength difference between input Rayleigh and converted S-waves suggests that the wavefront is mainly reflected upward in the bulk according to Snell’s law only partly converting into backward travelling Rayleigh waves along the bottom surface. In the P conversion case, the effect is exacerbated and differences in wavelength, velocity and propagation direction between top and bottom signal are clear.

To conclude, we have shown that crystal momentum transfer via Umklapp scattering is of paramount importance to furthering the modalities of many metamaterial devices. Leveraging this decades old phenomenon with modern, advanced structured materials permits these remarkable devices to harness further powers of wave manipulation. For elasticity we have experimentally verified a tailored surface wave to body wave reversed conversion effect, which allows the distinct bulk wave-types to be separated at will (Supplementary Note 4, Supplementary Fig. 3). The incorporation of these mechanisms motivates hybrid effects with self-phased systems, with potential to spark new paradigms of control across all wave regimes.

## Methods

### Fabrication

The aluminium resonators were printed by selective laser melting (SLM). The Rayleigh wave was generated by a Panametrics Videoscan V414 0.5 MHz plane wave transducer and coupled into the sample by a 65° polymer wedge. Phenyl salicylate was used to glue the transducer and wedge to the sample providing good coupling and long-term stability. A Ritec RPR-4000 programmable pulser drove the transducer using a three-cycle sinusoidal burst at 1 and 1.5 MHz for S and P-conversion, respectively, with an amplitude of 300 V peak-to-peak and repetition rate of 500 Hz. At this repetition rate, there were no echoes from previous pulses. The sample was mounted on scanning stages and measured with a rough-surface capable optical detector (Bossanova Tempo-10HF) over an area of 100 × 30 mm and we used a 0.25 mm step-size. An Agilent digital oscilloscope was used to captured the signal with 125 MSa s^−1^ and 512 averages before transfer to a desktop computer.

### Simulation

The 2D wavefield in the halfspace characterised by Rayleigh, P and S waves is simulated using SPECFEM2D, a code that solves the elastic wave equation using a second-order Newmark integration scheme in time and the spectral element method in space. The code is parallelised using domain decomposition and the MPI instruction set. The quadrilateral mesh is created using the commercial software Trelis. Stress-free conditions are applied to the rod’s boundary and the top surface, while the lateral and bottom borders are supplied with absorbing boundaries to avoid undesired reflections. The code runs on a parallel cluster on 32 CPUs. A typical simulation for this work runs in <5 min. 2D and 3D figures have been created using Python library Matplotlib, Matlab and Blender. Independent corroborations using Abaqus opposed to SPECFEM2D are presented (Supplementary Note 1).

## Supplementary information

Supplementary Information

Description of Additional Supplementary Files

Supplementary Movie 1

Supplementary Movie 2

Supplementary Movie 3

Supplementary Movie 4

## Data Availability

The data that supports the findings of this study are available from the corresponding author upon reasonable request.

## Code availability

The software SPECFEM2D is available as an open source code at: https://geodynamics.org/cig/software/specfem2d/ Meshing and configuration files for the simulations presented here can be obtained upon reasonable request from Dr. Colombi (colombia@ethz.ch).
