# Supplementary material for: Tailored elastic surface to body wave Umklapp conversion
Source: Nat Commun. 2020 Jun 29;11:3267. doi: 10.1038/s41467-020-17021-x (PMC7324571; doi:10.1038/s41467-020-17021-x)
Supplement: Supplementary file 1 — Supplementary Information [file 41467_2020_17021_MOESM1_ESM.pdf]

Tailored elastic surface to body wave Umklapp conversion: Supplementary Information

Chaplain et al.

Tailored elastic surface to body wave Umklapp conversion: Supplementary Information

Gregory J. Chaplain[1], Jacopo M. De Ponti[2,3], Andrea Colombi[4], , Rafael Fuentes-Dominguez[5], Paul Dryburg[5], Don Pieris[5], Richard J. Smith[5], Adam Clare[6], Matt Clark[5] and Richard V. Craster[1,7]

[1,*] *Department of Mathematics, Imperial College London, London SW7 2AZ, UK*

[2] *Dept. of Civil and Environmental Engineering, Politecnico di Milano, Piazza Leonardo da Vinci, 32, 20133 Milano, Italy*

[3] *Dept. of Mechanical Engineering, Politecnico di Milano, Via Giuseppe La Masa, 1, 20156 Milano, Italy*

[4] *Dept. of Civil, Environmental and Geomatic Engineering, ETH, Stefano-Franscini-Platz 5, 8093 Zürich, Switzerland*

[5] *Optics and Photonics, Faculty of Engineering, University of Nottingham, Nottingham, NG7 2RD, UK*

[6] *Advanced Component Engineering Laboratory (ACEL), Faculty of Engineering, University of Nottingham, NG7 2RD, Nottingham, UK*

[7] *Department of Mechanical Engineering, Imperial College London, London SW7 2AZ, UK*

[*] *Corresponding author: gregory.chaplain16@imperial.ac.uk*

Supplementary Note 1:

To further elucidate the insight underlying how the reversed conversion effect is achieved, we present a detailed analysis of the full dispersion curves along with carefully chosen additional examples of the reversed conversion effect; experimental details are also provided. As an additional independent validation, scattering simulations were performed using the finite element software Abaqus [1] (not presented), as opposed to SPECFEM used within the main article.

We emphasise that Eq. (1), presented in the main text, does not violate momentum conservation [2]; unlike for conventional graded structures we are not considering only the true momenta of interfering waves (phonons) within a crystal, but taking advantage of the momentum of the system as a whole. To utilise Umklapp U-processes for surface waves interacting with a graded structure with constant periodicity between resonant elements, it is paramount to obtain the dispersion curves for a perfectly periodic, infinite array of rods of fixed height; the adiabatic grading of the array allows the dispersion curves at each grading parameter to be used to infer the behaviour of the entire array; this assumption is now commonplace in the design of graded/chirped structures [3, 4].

Supplementary Note 2: Dispersion curves

In Supplementary Figure 1 we show a portion of the extended dispersion curves, up to the edge of the second Brillouin zone, marked X', for a perfectly periodic medium of resonant rods atop an elastic half space at fixed height $h = 0.5$mm where the diameter of the rods and periodicity of the array are given in Supplementary Table 1. Using COMSOL multiphysics finite element software these dispersion curves, along with the relative rod motion (either flexural or longitudinal), are calculated. The rod's dominant behaviour is important in terms of how the surface wave hybridises into a bulk wave, and is shown through the colormap of the dispersion curves, with purple denoting longitudinal (axial) motion and blue displaying flexural motion. Conventionally when considering surface waves in periodic graded systems, only the lowest two modes below the Rayleigh line are considered [3, 5]; these modes are clearly seen in Supplementary Figure. 1(a) as they are strongly localised to the surface and therefore are clearly identifiable. For higher modes, whose frequencies correspond to wavevectors outside the first Brillouin Zone (BZ), the dispersion curves for surface waves are more difficult to ascertain, due to the large number of spurious solutions [6] found by the eigenvalue solver; these arise from the finite depth of the simulated region and correspond to propagating modes or modes created by the finite layer. The relative degree of longitudinal/flexural motion for higher modes is not as clear as for the lowest modes. To extract the excited, localised surface modes presented in Figure 2(c), we use a weighted averaging technique to interpolate, and hence separate, both motions independently. We remove curves with the opposite behaviour below a set tolerance on the vertical and horizontal displacements of the rods; the ellipses above the rods in Fig. 3 represent this ratio. At each wavevector, the remaining modes are then averaged with a weight normalised to the degree of desired behaviour. Polynomial interpolation of the subsequent curves then gives the estimated higher order modes, that are extracted and presented independently of the spurious solutions, as in Figure 2 (for the second order longitudinal mode). As an example, the second (averaged) longitudinal mode is shown by the dotted black line in Figure 1, corresponding to that of the first rod in Figure 2(c) in the main text.

Showing the dispersion curves up to the edge of the second BZ, as we do in Supplementary Figure 1, highlights an important nuance when dealing with periodic media.

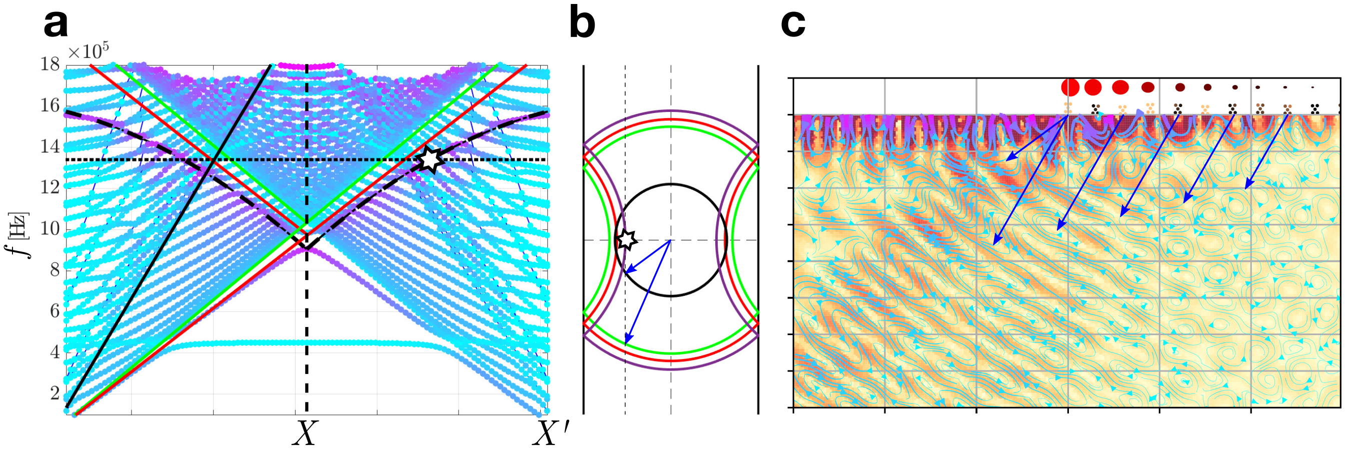

Supplementary Figure 1. **Extended Dispersion curves and ungraded arrays.** (**a**) A portion of the extended dispersion curves for an array of rods with parameters as in Supplementary Table 1. The Rayleigh, shear and compressional sound lines are shown in red, green and black respectively. The relative dominance of longitudinal to flexural rod motion is shown by the colormap with purple/blue being predominantly longitudinal/flexural. Dashed black lines show the interpolated second longitudinal mode, with excitation frequency of 1.34MHz used in Fig. 2 as the dotted horizontal line. Band folding occurs so that the IBZ is shown up to the dashed vertical black line. The excited mode wavevector is highlighted by the white star that, after the transfer of crystal momentum is reversed and the scattered wavevector is shown in the corresponding isofrequency contours in (**b**). Panel (**c**) shows scattering by an ungraded array consisting of perfectly periodic array of resonators, as defined in Supplementary Table 1, with $\Delta h = 0$, excited by an incoming Rayleigh wave of frequency 1.34MHz. Umklapp scattering takes place without grading, but is not confined to a beam since no effective band gap reached; this is illustrated by the eccentricity of the ellipses above the rods remaining unchanged. A mixture of shear (S) and compressional (P) waves is produced as predicted from the isofrequency curves in (b).

When considering the band structures of periodic media it is conventional to plot the dispersion curves within the Irreducible Brillouin Zone (IBZ). This is a physically significant representation of the band structure which compactly contains all essential information within an interval in k-space defined by the symmetries within the periodic structure [7]. There are certain dangers of using this representation however [8], particularly when considering its construction through the use of 'band folding'; the IBZ is formed by virtue of the fact that the wavevectors in a periodic system are defined modulo a reciprocal lattice vector. That is, wavevectors $\boldsymbol{\kappa}$ and $\boldsymbol{\kappa}' = \boldsymbol{\kappa} - \boldsymbol{G}$ are equivalent. As such, when using the terminology of band folding in the construction of the IBZ, one may expect that the excited wave vector highlighted by the star in Supplementary Figure 1 corresponds to an excited mode at the position reflected (or folded) about the BZ edge at X, i.e. to the left of the BZ boundary. However, this mode is actually equivalent to the highlighted mode by the star translated by a reciprocal lattice vector $\boldsymbol{G}$, which lies in the region of k-space to the left of the origin Γ, such that $-\pi/a < \kappa < 0$, or $-X < \kappa < \Gamma$. It is exactly by the transfer of crystal momentum (i.e. Umklapp scattering) that this mode is reached within the graded array when excited by an incident Rayleigh wave with wavevector outwith that of the first BZ. We highlight this to emphasise that care should be taken when visualising the band structure through a folding argument.

For a given frequency of excitation, a transition in the dominant behaviour of the rod motion can be engineered at a selected spatial position by the grading of the array. The introduction of a grading parameter, for example the rod height, can result in an effective bandgap where there is a change in preferential dominance of the rods. It is important to note that this is not a true bandgap resulting from the periodicity or symmetry breaking. Despite this, at the position where the rod's dominant behaviour changes, U-processes dominate and a confined reverse converted beam is obtained. The confinement of the beam of the reversed body wave is dictated by the grading parameter.

In the main text we show a compact diagram which allows the prediction of the angle of the reversed conversion (Figure 2(c)). This is a superposition of the projections of the simplified contours of the system into one plane; despite being a 1D periodic system, we show isocircles to aid visualisation and prediction of the angles of the reversed conversion. Supplementary Figure 2 shows a detailed description of this procedure.

Diameter, t	periodicity, a	initial height, h_0	grading, Δh
0.5mm	1.5mm	0.5mm	0.05mm

Supplementary Table I. **Array, and rod, parameters used in simulations and experiments**

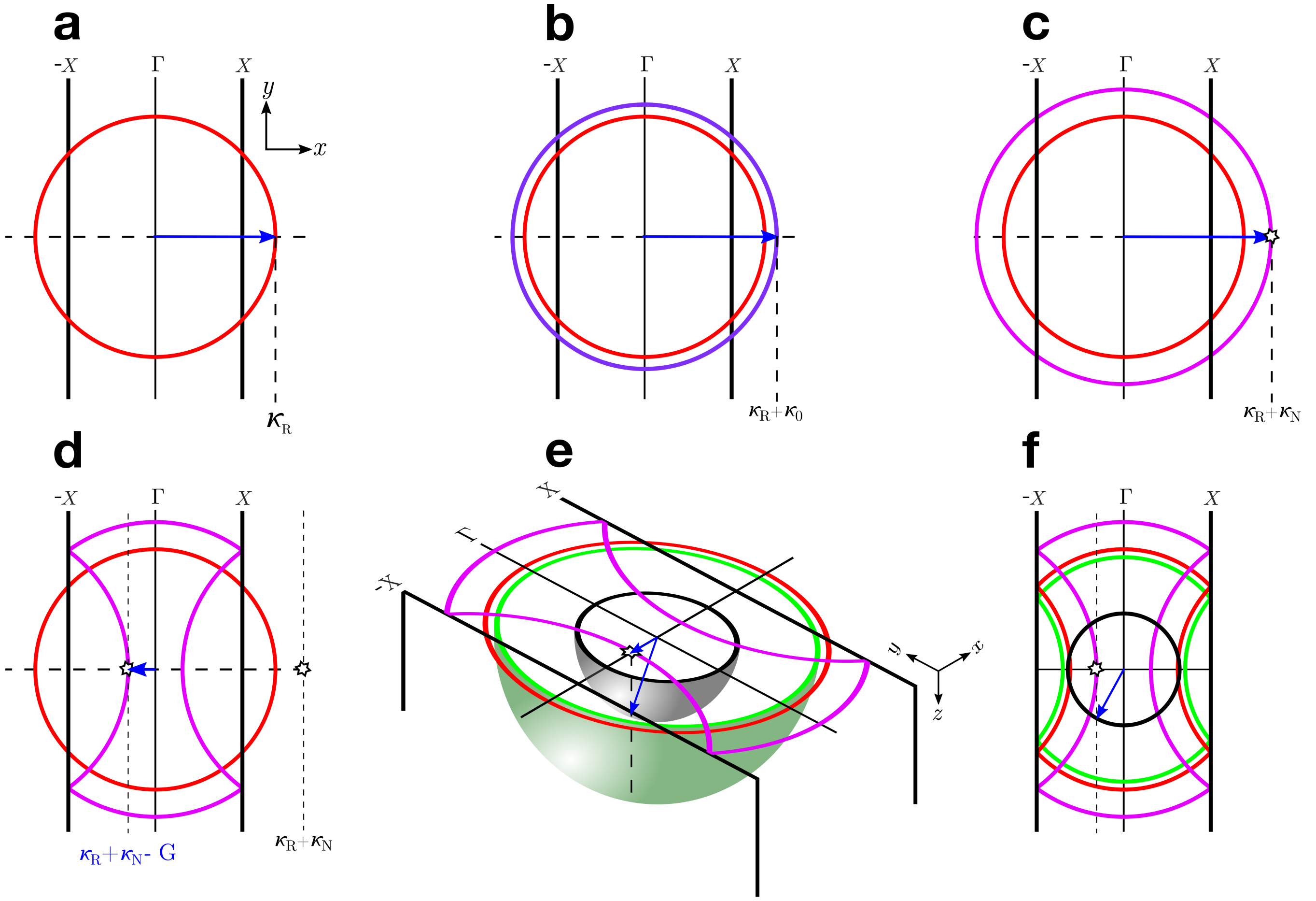

Supplementary Figure 2. **Predicting the angle of conversion.** To determine the angles and polarisation of the reversed conversion effect we analyse the incident wavevector, and how it is altered, relative to the first BZ of the periodic array, for the dispersion curves presented in Figure 2. We draw the 1D BZ of the periodic array as an infinite strip, allowing us to relate the contours of the bulk and surface waves of the free material to the array. (**a**) shows the isofrequency contour of a surface Rayleigh wave in the $x-y$ plane, incident on the array. Since this is excited in the free, isotropic surface this is indeed an isocircle. This defines the incident Rayleigh wavenumber, κ_R, marker by the blue arrow. Upon reaching the array, array guided waves are excited with wavenumber spatially determined by the dispersion curves for the perfectly periodic medium with a given rod height. For the first encountered rods the incident Rayleigh wave excites a guided wave with wavevector $\kappa_R + \kappa_0$. (**b**) shows this change in wavenumber for a perfectly periodic array of rods of height h_0; only the x component is present since we have a 1D periodic structure, but we draw a 'contour' at this wavevector to aid explanation, with colour that matches the corresponding rod in Figure 2. Since the array is graded adiabatically there is a smooth transition in wavenumber as the wave propagates along the array, until an effective bandgap is reached. (**c**) shows the last supported wavevector, at the Nth rod, $\kappa_R + \kappa_N$. Since the resultant wavevector is in the second BZ of the locally periodic structure, the transfer of crystal momentum (Umklapp scattering) takes place. (**d**) shows the translated, or flipped, wavevector by a reciprocal lattice vector $\boldsymbol{G} = 2\pi/a$, with a the periodicity. It is not clear at this stage that any mode conversion will take place; only the $x-y$ surface waves have been inspected so far. To rectify this (**e**) shows the isosurfaces of the body waves in the z direction, relative to the first BZ. These are half-spheres as the bulk material is homogeneous. Then, by conserving the tangential component of the reversed wavevector in this 3D picture we can see which, if any, of the shear and compressional isosurfaces are intersected. This gives us the prediction and polarisation of the reversed body wave. (**f**) shows a compactified version of (**e**), projecting all contours into the same plane with the folding of the contours of the free space waves to lie within the first BZ, even though they exist independently of the periodic material. This allows the angle in the z direction to be seen in the same plane as the incident surface wave contours.

Supplementary Note 3: Ungraded arrays

As detailed in the main text, Umklapp scattering occurs independently of any grading parameters, so long as the frequencies of excitation result in wavevectors that are outwith the first BZ. This has been shown in electromagnetic systems between regions of abrupt changes in periodicity [9]; these are not required in the elastic system since surface Rayleigh waves exist at all frequencies independent of any structuring. In light of this we show that

Umklapp scattering generates the reversed conversion effect for a single ungraded array of rods, for the same configuration as in Supplementary Table 1, but with $\Delta h = 0$. Supplementary Figure 1(b) shows the isofrequency contours of an incident wave at frequency 1.34MHz in purple (obtained from the averaged longitudinal dispersion curves), with the isocircles of the Rayleigh, shear and compressional lines in red, green and black; these are not all defined in the same plane (Rayleigh waves do not propagate in the bulk, and indeed for 1D arrays are points on the horizontal wavenumber axis and S/P waves cannot be localised to the surface), but for clarity we show them superimposed, and extended to circles, to aid the explanation. Supplementary Figure 1(c) shows the result of a scattering simulation with no change in resonator parameters: Umklapp scattering is clearly seen, with a mixture of both S and P waves, since there is no effective band gap to confine the conversion into a confined beam of a single wave type. A mixing of polarisations in the reverse converted wave is present due to there being no distinct preferential rod polarisation (the ratio of the flexural and longitudinal response of the rod traces out circles rather than ellipses as in Figure 3). This is highlighted in the reverse scan experimental results (Supplementary Figure 6), where the distinction in excited wavelength on the bottom surface is not as pronounced as the clear separation in P and S polarisations in Figure 1. The amplitude of the localised wave is reduced as it transits the array as a result of crystal momentum transfer at every point along the array and this then forms a leaky elastic antenna waveguide.

Supplementary Note 4: Pure P-conversion

To exemplify the unprecedented tailored control achieved by simple metawedge structures we demonstrate mode conversion of the surface wave into exclusively a P-wave in the bulk. This is achieved using the graded array presented in the main article, described by the parameters in Supplementary Table 1, but now with the frequency increased to 1.7MHz such that the effective band gap is reached almost immediately along the array. A SPECFEM simulation of this scenario is shown in Supplementary Figure 3 whereby, due to the increase in frequency, the resulting reverse converted compressional body wave is produced at nearly 90° from the array axis. We have therefore achieved separation of the compressional body waves from any shear waves produced by scattering from the interface, and also been able to tune the angle of conversion.

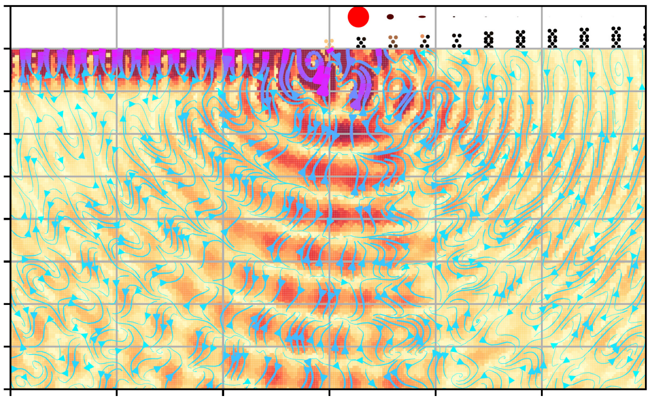

Supplementary Figure 3. **Pure P Conversion.** Surface Rayleigh wave to body P-wave conversion directed at 90° from the array, described in Supplementary Table 1, excited at 1.7MHz. The separation of the compressional and shear wave-types is clearly visible from the streamlines.

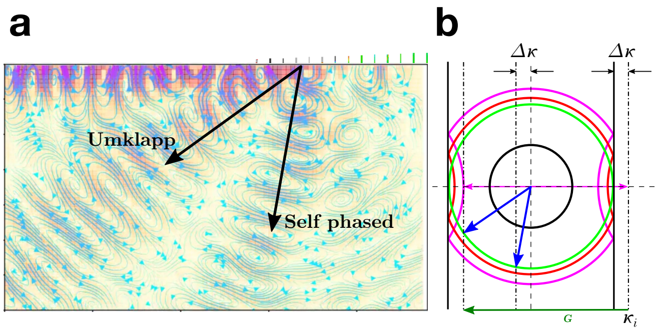

Supplementary Figure 4. **Combining Umklapp and self-phased arrays for double S-wave reversed conversion.** (**a**) shows the wave-type of the body wave through the streamlines, whilst (**b**) combines the Umklapp analysis with that of [10]. The incident surface wave has wavenumber κ_i and $\Delta\kappa = |\kappa_i - \pi/a|$ is the difference between it and the wavenumber at the band-edge. The grading is chosen such that the self-phased effect takes place before the effective band gap and cut-off position are met.

Supplementary Note 5: Combining Umklapp and Passive self-phasing

In [10], for a simpler scalar problem, we developed a passive self-phasing array using graduations of a structured line array. In this section we combine this with the Umklapp mechanism. Supplementary Figure 4 shows the quite remarkable splitting of the incident surface wave, where we have an incident array vector of κ_i (of frequency 1.04MHz), into *two* reversed S-body waves.

The self-phasing effect arises due to the propagation of the surface wave through the graded region until the effective bandgap is reached and the surface wave then slows down and stops. Once the phase has been altered, such that, in reciprocal space, the frequency and wavenumber are at the band-edge reflection then occurs; upon reflection, the wave is endowed with a phase change of $-\pi/a$, with a being the periodicity.

This reversed surface wave then undergoes similar phase changes as it transverses the array in the oppo-

site direction. Viewing this series of phase changes in isofrequency phase space, as shown in Supplementary Figure 4(b), elucidates that the initial κ_i wave vector is reflected and translated a distance $-\Delta\kappa$ from the origin, and this then co-exists with the U-processes (for which there is a phase shift of $2\pi/a$) resulting in the second reversed shear wave, at a different angle.

Despite the resultant vector lying within the isocircle of the compressional body wave, see Supplementary Figure 4(b), the shear mode is preferentially excited by virtue of the matching between the flexural motion of the rods and the shear waves. The self-phasing effect is intrinsically different to the use of Umklapp scattering; for U-processes we require the transfer of crystal momentum, whereas for passive self-phased arrays we utilise reflection at the band edge. If the grading is designed accordingly, i.e. both rod height and array periodicity altered, the two effects can be separated in space. U-processes take place along the array until the effective band gap is reached (Supplementary Figure 1), and so there is still propagation of energy along the array. It is in this hybrid region where self-phasing can be utilised.

Supplementary Note 6: Reversing the grading profile

A natural question is how reversing the orientation of the graded array in the main text affects the results, that is, going from tall to short resonators rather than vice-versa. We present here the analysis and experimental results for this case. For these rod heights, there is considerable mixing between the longitudinal and flexural motion of the rods, and there is no distinct separation between longitudinal and flexural rod motions and as a result no clear effective band gap is created. As a result, the wave propagates along the array, experiencing U-processes at every position, since the operational frequencies are still within the second BZ (only the rod heights are changing, not the periodicity). Therefore similar effects to those in the main article occur, except that the reversed Umklapp conversion does not result in a well confined beam; the array behaves as a leaky antenna waveguide, similarly to the ungraded array of Supplementary Figure 1.

Supplementary Figure 5 allows us to compare and contrast the response as we alter the orientation of the array relative to the incident surface wave. Here we show comparisons of the logarithms of the amplitudes of the Fourier components measured along the array axis. In the main text we discuss the situation of the surface wave incident upon an array graded from short to tall resonators; Supplementary Figure 5(a,c) shows this case for P and S reversed conversion confirming the discussion and interpretation of the main article; there is a sharp conversion from surface wave to body wave (as seen by its impact upon the bottom surface).

For the opposite case of the wave travelling from tall to short resonator heights, this sharp conversion does not occur and energy is carried to the end of the array, with the conversion effect occuring along the array; an effective bandgap is not reached as there is no clear distinction between longitudinal and flexural motion for the taller rods, shown in Supplementary Figure 6 (the counterpart of Figure 1 of the main text). By inspecting the dispersion curves associated with each local rod height the prediction of the angle of reversal is then dictated by the local Umklapp reversed wavevector and the sharp conversion is replaced by a distributed leakage of energy into reversed waves.

SUPPLEMENTARY REFERENCES

[1] Abaqus, G. Abaqus 6.11. *Dassault Systemes Simulia Corp Providence, RI, USA* (2011).

[2] Peierls, R. Zur kinetischen theorie der wrmeleitung in kristallen. *Annalen der Physik* **395**, 1055–1101 (1929).

[3] Colombi, A., Colquitt, D., Roux, P., Guenneau, S. & Craster, R. V. A seismic metamaterial: The resonant metawedge. *Sci. Rep.* **6**, 27717 (2016).

[4] Romero-García, V., Picó, R., Cebrecos, A., Sánchez-Morcillo, V. J. & Staliunas, K. Enhancement of sound in chirped sonic crystals. *Appl. Phys. Lett.* **102**, 091906 (2013).

[5] Colombi, A. *et al.* Enhanced sensing and conversion of ultrasonic Rayleigh waves by elastic metasurfaces. *Sci. Rep.* **7**, 6750 (2017).

[6] Chaplain, G., Makwana, M. & Craster, R. Rayleigh-Bloch, topological edge and interface waves for structured elastic plates. *Wave Motion* **86**, 162–174 (2019).

[7] Meyers, H. & Myers, H. *Introductory solid state physics* (CRC press, 1997).

[8] Craster, R. V., Antonakakis, T., Makwana, M. & Guenneau, S. Dangers of using the edges of the Brillouin zone. *Phys. Rev. B* **86**, 115130 (2012).

[9] Chaplain, G. J. & Craster, R. V. Ultrathin entirely flat umklapp lenses. *Phys. Rev. B* **101**, 155430 (2020).

[10] Chaplain, G. J. & Craster, R. V. Flat lensing by graded line meta-arrays. *Phys. Rev. B(R)* **99**, 220102 (2019).

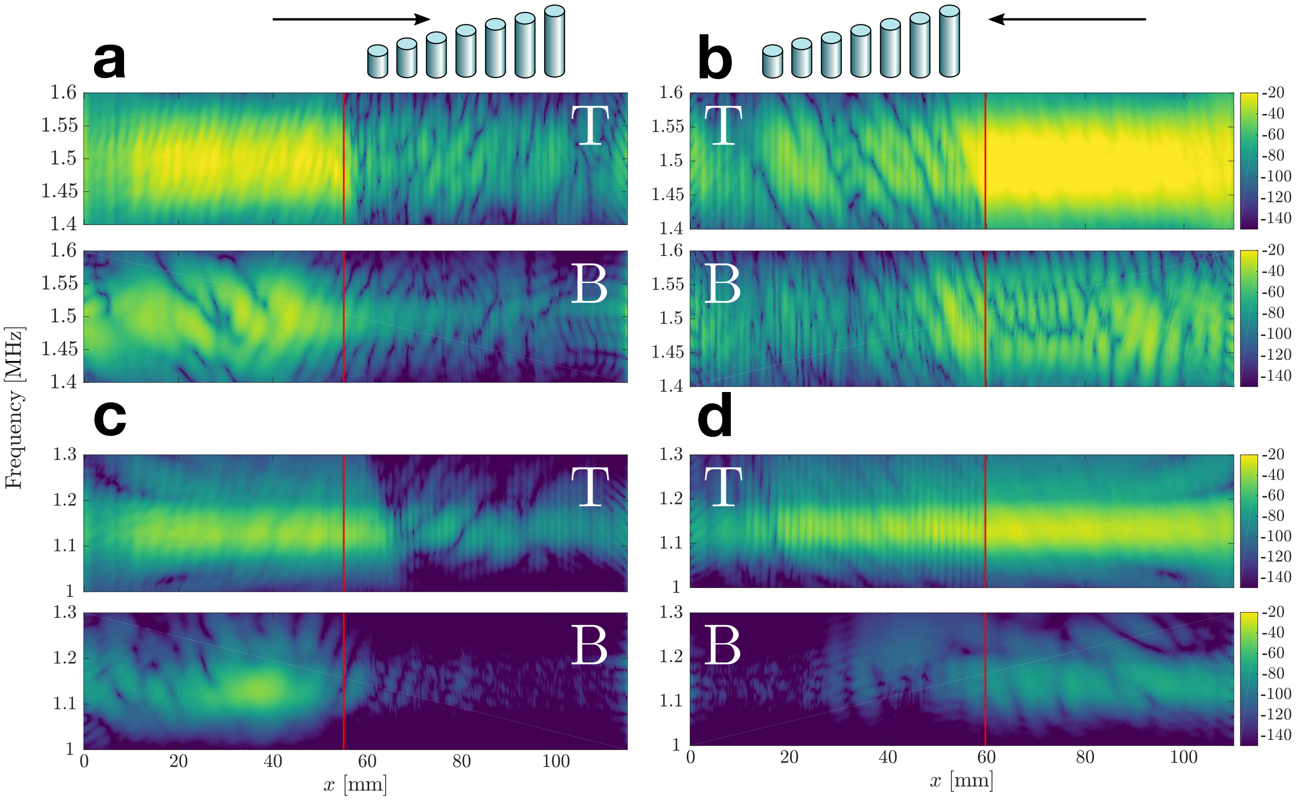

Supplementary Figure 5. **Fourier Analysis.** Logarithm of amplitude of Fourier components, extracted from experiments, as function of horizontal position on top and bottom surfaces, marked T,B respectively. Shown above each column is the direction of wave propagation and grading direction of the rods, with array starting from solid red line. (**a**,**b**) Show reversed P wave conversion with (**c**,**d**) showing reversed S wave conversion, for filtered frequencies between $1.45 - 1.55$MHz and $1.1 - 1.2$MHz respectively. In each case there is a clear reduction in amplitude as the wave on the top surface is reflected and reverse converted by the array. The effect of propagating from short to tall resonator height shows the effective band gap confines the reversed beam compared to exciting from tall to short.

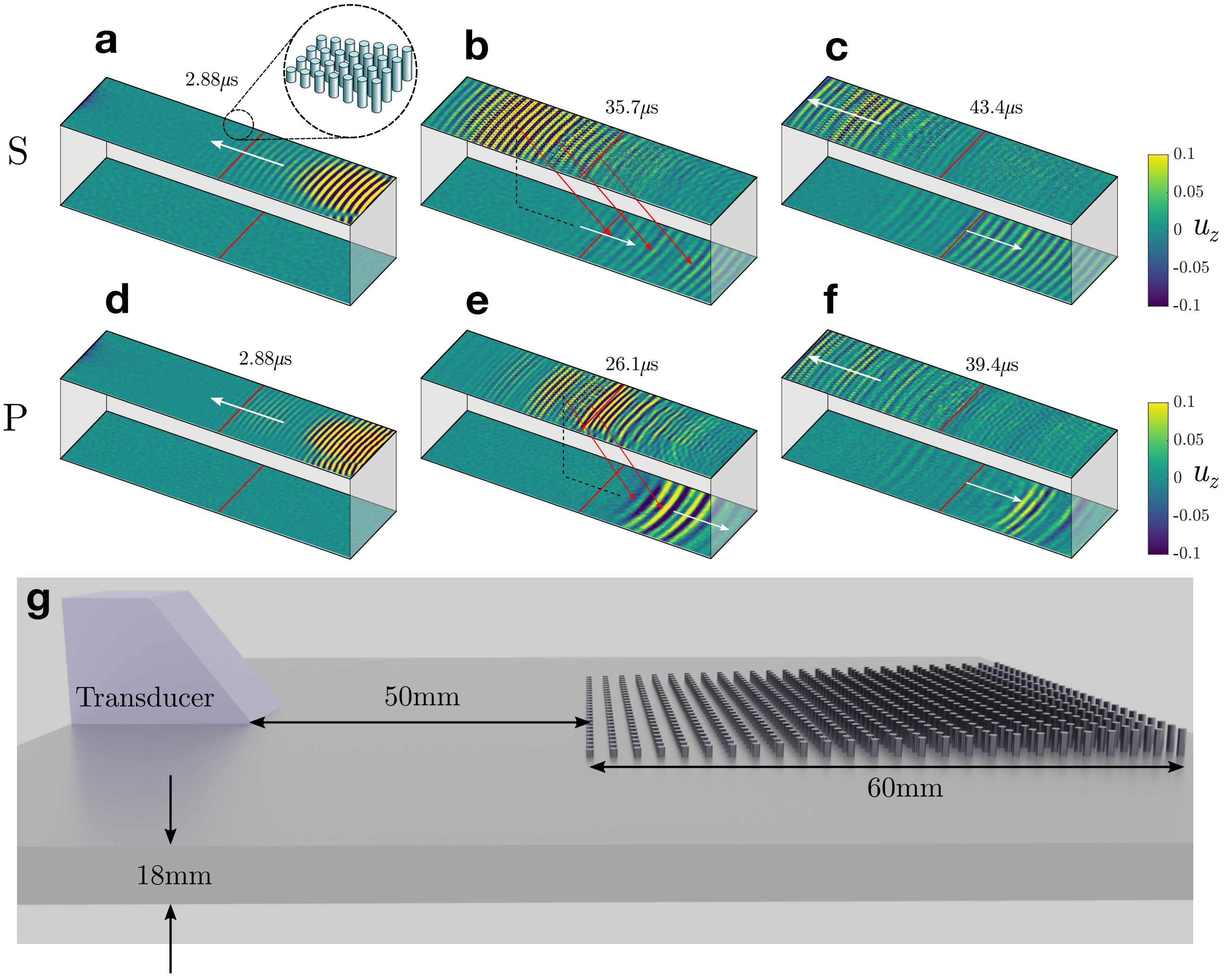

Supplementary Figure 6. **Backwards Scan Experimental results.** snapshots of temporal-spatial filtered scans from tall resonators to short resonators (i.e. a reversed excitation direction compared to that in the main text) along top and bottom surfaces observing S conversion (top row) and P conversion (bottom row), filtered between $1.1 - 1.2$MHz and $1.45 - 1.55$MHz respectively. Solid red lines show position where graded array begins, with zoom on the array geometry. Shown too is a schematic of the arrangement between the transducer and the array, with the plate width.
