# Supplementary material for: Tailored elastic surface to body wave Umklapp conversion
Source: Nat Commun. 2020 Jun 29;11:3267. doi: 10.1038/s41467-020-17021-x (PMC7324571; doi:10.1038/s41467-020-17021-x)
Supplement: Supplementary file 2 — Description of Additional Supplementary Files [file 41467_2020_17021_MOESM2_ESM.pdf]

Description of Additional Supplementary Files

File name: Supplementary Movie 1
Description : Filtered experimental data showing reversed P conversion in the forward direction (rod height increasing in direction of propagation), corresponding to Fig. 1(d-f).

File name: Supplementary Movie 2:
Description: Filtered experimental data showing reversed S conversion in the forward direction (rod height increasing in direction of propagation), corresponding to Fig. 1(a-c).

File name: Supplementary Movie 3:
Description: Filtered experimental data showing reversed P conversion in the backwards direction (rod height decreasing in direction of propagation), corresponding to Supplementary Fig. 6(d-f).

File name: Supplementary Movie 4:
Description: Filtered experimental data showing reversed S conversion in the backwards direction (rod height decreasing in direction of propagation), corresponding to Supplementary Fig. 6(a-c).
